# Impact of Censored or Penalized Data in the Genetic Evaluation of Two Longevity Indicator Traits Using Random Regression Models in North American Angus Cattle

**DOI:** 10.3390/ani11030800

**Published:** 2021-03-12

**Authors:** Hinayah R. Oliveira, Stephen P. Miller, Luiz F. Brito, Flavio S. Schenkel

**Affiliations:** 1 Centre for Genetic Improvement of Livestock, Department of Animal Biosciences, University of Guelph, Guelph, ON N1G 2W1, Canada; schenkel@uoguelph.ca; 2 Department of Animal Sciences, Purdue University, West Lafayette, IN 47907, USA; britol@purdue.edu; 3 Angus Genetics Inc., Saint Joseph, MO 64506, USA; SMiller@angus.org

**Keywords:** beef cattle, missing record, penalty method, productive life, stayability, survival

## Abstract

**Simple Summary:**

Cow longevity is a key driver of the beef cattle industry profitability that can be improved through genetic and genomic selection. Censored data are commonly dealt with in genetic evaluations of longevity, which can unfavorably impact the accuracy of breeding values and the rates of genetic progress. In this study, we evaluated alternative scenarios to define the best approaches for genetically evaluating longevity in North American Angus cattle in the presence of censored data.

**Abstract:**

This study aimed to evaluate the impact of different proportions (i.e., 20%, 40%, 60% and 80%) of censored (CEN) or penalized (PEN) data in the prediction of breeding values (EBVs), genetic parameters, and computational efficiency for two longevity indicators (i.e., traditional and functional longevity; TL and FL, respectively). In addition, three different criteria were proposed for PEN: (1) assuming that all cows with censored records were culled one year after their last reported calving; (2) assuming that cows with censored records older than nine years were culled one year after their last reported calving, while censored (missing) records were kept for cows younger than nine years; and (3) assuming that cows with censored records older than nine years were culled one year after their last reported calving, while cows younger than nine years were culled two years after their last reported calving. All analyses were performed using random regression models based on fourth order Legendre orthogonal polynomials. The proportion of commonly selected animals and EBV correlations were calculated between the complete dataset (i.e., without censored or penalized data; COM) and all simulated proportions of CEN or PEN. The computational efficiency was evaluated based on the total computing time taken by each scenario to complete 150,000 Bayesian iterations. In summary, increasing the CEN proportion significantly (*p*-value < 0.05 by paired *t*-tests) decreased the heritability estimates for both TL and FL. When compared to CEN, PEN tended to yield heritabilities closer to COM, especially for FL. Moreover, similar heritability patterns were observed for all three penalization criteria. High proportions of commonly selected animals and EBV correlations were found between COM and CEN with 20% censored data (for both TL and FL), and COM and all levels of PEN (for FL). The proportions of commonly selected animals and EBV correlations were lower for PEN than CEN for TL, which suggests that the criteria used for PEN are not adequate for TL. Analyses using COM and CEN took longer to finish than PEN analyses. In addition, increasing the amount of censored records also tended to increase the computational time. A high proportion (>20%) of censored data has a negative impact in the genetic evaluation of longevity. The penalization criteria proposed in this study are useful for genetic evaluations of FL, but they are not recommended when analyzing TL.

## 1. Introduction

Angus is the most common beef cattle breed currently raised in the United States [1,2], the top beef cattle producer in the world [3]. Longevity was identified by North American beef cattle stakeholders as the utmost priority for further genetic improvement [4]. Genetic (and genomic) selection for longevity has the potential to reduce the costs associated with the replacement of animals in the herd and, depending on the trait definition assumed for longevity in the breeding program, it can also improve the reproductive potential of mature cows [5,6]. Moreover, selecting for improved longevity can contribute to increased genetic progress for other economically important traits, as it can increase the number of animals available for selection (i.e., greater selection intensity for other traits). The potential increase in the number of animals in the herd is mainly due to the fact that less animals are culled due to involuntary reasons, such as disease and structural problems [7].

Genetic selection for increased longevity can be challenging as this is a trait measured late in life and many cows are still alive at the time of the genetic evaluation [8], or because their culling might not be recorded or informed to the breeding program [7]. Thus, the use of censored data is commonly dealt with in the genetic evaluations of longevity. In this context, Hou et al. [9] showed that merely excluding phenotypes from the genetic analysis can lead to bias, especially for sires that have a high proportion of daughters with censored records. Furthermore, eliminating records from cows that are still alive can contribute to reducing the accuracy of breeding values (EBVs) predicted for them and their parents, which can considerably reduce the genetic gain per unit time because older animals (which tend to have lower genetic merit compared to younger animals in a population under selection) might continue to be selected.

The penalty method [10] has been considered suitable to treat censored records in the genetic evaluations of fertility related traits, which consists on applying a penalization criterion for animals with censored records. For age at first calving, for example, censored records are replaced by a set of augmented data (i.e., penalized data), which is obtained by adding a constant value of 21 days to the highest value of age at the first calving within each contemporary group [9,10,11]. Specifically for longevity-related traits, penalized data can be obtained by including a culling date for cows without culling information recorded in the dataset. In this context, the last calving information available for each cow can be used to define the penalization criterion for longevity. For instance, the culling data for each cow can be assumed anytime (e.g., one or two years) after its last reported calving. Therefore, the greatest challenge is to defined the optimal threshold to create the penalized data for longevity. Using the augmented data instead the censured records has been shown to increase the prediction accuracy of EBVs for fertility in some studies [11,12]. However, to our best knowledge, the use of the penalty method has not yet been investigated for the genetic evaluation of longevity indicator traits, especially when using random regression models (RRM).

Accounting for censored records in the genetic evaluation is simpler when using RRM, because they do not require that all animals have information for all time points [13,14]. In addition, the use of RRM usually results in more accurate EBVs compared to other statistical models, and it might allow the identification of the most feasible time periods to perform selection [13,15]. Recently, the optimal RRM to perform selection for longevity in North American Angus cattle was defined, while comparing the impact of different longevity indicators in the selection scheme [6]. However, the impact of censored or penalized data in the selection decisions remains unknown when performing genetic evaluations for longevity using RRM. In this context, we aimed to evaluate the impact of different proportions of censored data (i.e., 0%, 20%, 40%, 60%, and 80%) in the prediction of EBVs, genetic parameters, and computational efficiency for two longevity indicators proposed for North American Angus cattle [6]: (a) traditional longevity (TL; defined as the time from first calving to culling); and (b) functional longevity (FL; defined as the time period in which the cow was alive and calving after its first calving). Additionally, three different criteria for the penalty method were proposed and extensively compared to the use of censored records.

## 2. Materials and Methods

### 2.1. Ethics Statement

All information used in this study was obtained from existing datasets provided by the American Angus Association (Saint Joseph, MO, USA) and the Canadian Angus Association (Rocky View County, AB, Canada). Therefore, no animal care committee approval was necessary.

### 2.2. Dataset, Quality Control, Designs, Scenarios, and Sub-Scenarios

Only cows with information of natural death (i.e., cows that died due to natural causes or were culled after they were 15 years old) were analyzed in this study. The phenotypic quality control excluded data from cows born before 1990 or that did not have their first calving before 30 months-old, as well as cows with culling age greater than 20 years. Additional details about the original dataset and phenotypic quality control performed can be found in Oliveira et al. [6].

A total of 100,000 cows were randomly chosen from the 150,229 cows used in Oliveira et al. [6]. Thereafter, different proportions (i.e., 20%, 40%, 60%, and 80%) of cows were randomly assigned to have their culling information censored, which were used to create the sub-scenarios for censored data evaluated in this study. Cows that had their culling information censored in one sub-scenario were kept as censored in the next sub-scenario, in order to avoid any extra confounding effect. Therefore, an algorithm was created to randomly sample 20% cows and censor their records. Thenceforth, the algorithm would randomly sample 20% cows with uncensored records from each previous sub-scenario, in order to censor their records and create the next sub-scenario (with 40%, 60%, and 80% censored data, respectively). No constraints regarding the number of cows with censored/uncensored records per contemporary group were imposed, because contemporary groups (concatenation of herd-year-season) were assumed as random effects in this study.

The same sub-scenarios evaluated for censored data (in terms of proportion of censored/penalized data and cows sampled) were used to create the sub-scenarios for penalized data (i.e., a penalization criterion was used for cows that had their records previously censored). Three different criteria were proposed for the penalty method [10] used in this study. The first criterion assumed that all cows with censored records were culled one year after their last reported calving. The second criterion assumed that cows with censored records older than nine years were culled one year after their last reported calving, while censored records were kept for cows younger than nine years. Finally, the third criterion assumed that cows with censored records older than nine years were culled one year after their last reported calving, while cows younger than nine years were culled two years after their last reported calving. The threshold of nine years and the penalization of one or two years were defined based on the average culling age estimated for cows that died due to natural reasons [6], the number of cows culled per age (Supplementary Figure S1), and the proportion of reappearance in the dataset after one missing calving (Supplementary Figure S2).

To facilitate the comparison of results and subsequent discussion, hereafter scenarios based on the complete (i.e., 0% censored or penalized data), censored, and penalized data will be called COM, CEN, and PEN, respectively. The notation of the sub-scenarios is CEN20, CEN40, CEN60, and CEN80 to represent 20%, 40%, 60%, and 80% censored data, respectively. Similarly, the notation of the sub-scenarios used for PEN follows the pattern: PENxmy with x referring to the amount of penalized data and y to the penalty method used. For instance, PEN using 20% penalized data and the first penalty method (which assumes that all cows with censored records were culled one year after their last reported calving) was coded as PEN20m1.

**Figure 1:**
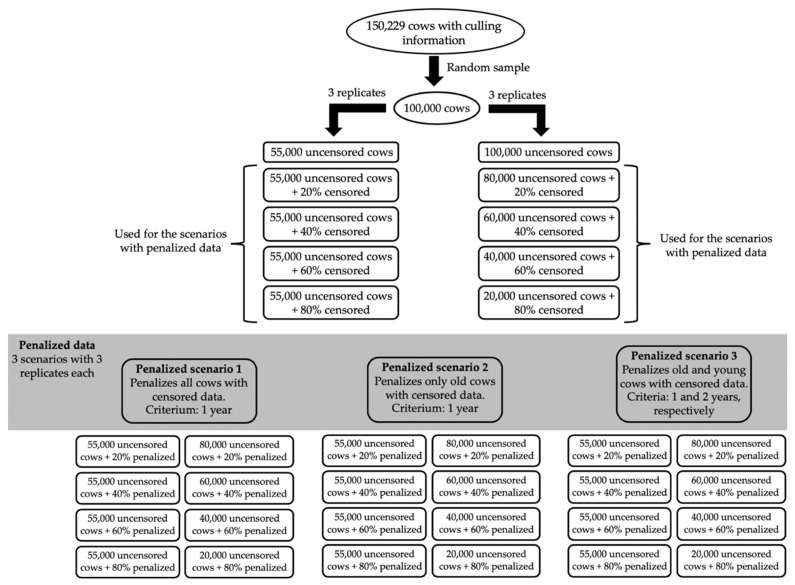
Scheme of the designs, scenarios, and sub-scenarios evaluated in the study. Grey area represents the scenarios using penalized data.

In order to avoid any statistical confounding between the number of animals and the number of censored/uncensored records in the results, two designs were used for each scenario/sub-scenario: (1) always including 55,000 cows with uncensored data; and (2) always including a total of 100,000 cows in the analyses. In this context, the first design considered a total of 55,000; 66,000; 77,000; 88,000; and 99,000 cows (i.e., 55,000 cows with uncensored records plus 0%, 20%, 40%, 60%, and 80% cows with censored or penalized records, respectively), while the second design considered always 100,000 cows, from which 100,000; 80,000; 60,000; 40,000; and 20,000 cows had uncensored data (plus 0; 20,000; 40,000; 60,000; and 80,000 cows with censored or penalized records, respectively). A total of three replicates were performed for each analysis, which differed according to the cows randomly sampled to have their records censored. The initial 100,000 cows were kept the same in all replicates. A scheme of the designs, scenarios and sub-scenarios used in this study is shown in Figure 1.

### 2.3. Longevity Indicators and Statistical Analyses

Two different longevity indicators were used to evaluate the impact of censored and penalized data in the prediction of EBVs, genetic parameters, and computational efficiency for the genetic analyses. For the first indicator, longevity was coded as 1 when the cow was alive, and 0 after the cow was culled (i.e., TL). For the second one, longevity was codified as 1 for cows that had calved at the specific age, 0 after the cow was culled, and as missing record when no information of calving was found at the specific age (i.e., FL). Both longevity indicators were evaluated from 2 to 15 years-old, using RRM. The RRM used in this study were defined as follows:

(1) y=Xb+Hq+Za+Wp+e,

in which **y** is the vector of observations, assumed as $y|b,q,a,p,R_{q},G_{0},R_{p},\sigma_{e}^{2}\sim N\left(Xb+Hq+Za+Wp,\;I\sigma_{e}^{2}\right)$; **b** is the vector of systematic effects (embryo transfer and coefficients of systematic regressions for year-season of birth), which was assumed as $b\sim N\left(0,\Sigma_{b}⊗I\right)$; **q** is the vector of random regression coefficients for the herd-year-season effects, which was assumed as $q|R_{q}\sim N\left(0,R_{q}⊗I\right)$; **a** is the vector of random regression coefficients for the animal additive genetic effects, which was assumed as $a|G_{0},\;A\sim N\left(0,G_{0}⊗A\right)$; **p** is the vector random of regression coefficients for the permanent environmental effects, which was assumed as $p|R_{p}\sim N\left(0,R_{p}⊗I\right);$ and **e** is the random vector of residuals, which was assumed as $e|\sigma_{e}^{2}\sim N\left(0,I\sigma_{e}^{2}\right)$. Fourth order Legendre orthogonal polynomials [16] were used for all regressions, as defined in a previous study using the same data [6].

The **X**, **H**, **Z**, and **W** are the incidence matrices for **b**, **q**, **a**, and **p**, respectively. In addition, $R_{q},\;G_{0},\;\mathrm{and}\;R_{p}$ are the herd-year-season, additive genetic, and permanent environmental variance components matrices, respectively. The matrices **A** and I are the pedigree-based additive relationship and the identity matrices, respectively. The matrix $\Sigma_{b}$ is a diagonal matrix with large variances (10^10^) to represent vague prior knowledge. The matrices $R_{q},\;G_{0},\;\mathrm{and}\;R_{p}$ were assumed to follow an inverted Wishart distribution (IW) with small prior knowledge, i.e., $R_{q}\sim \mathrm{IW}\left(3,{\hat{R}}_{q}\right)$, $G_{0}\sim \mathrm{IW}\left(3,{\hat{G}}_{0}\right)$, and $R_{q}\sim \mathrm{IW}\left(3,{\hat{R}}_{q}\right)$. A scaled inverted chi-squared distribution was assumed for $\sigma_{e}^{2}$.

Gibbs sampler based on the Markov Chain Monte Carlo (MCMC) algorithm was used to estimate the variance components and predict the breeding values for the regression coefficients using the Best Linear Unbiased Prediction (BLUP) method implemented in the gibbs3f90 software [17]. The MCMC chain length, burn-in, and thin used in this study were 150,000, 50,000, and 10, respectively. Convergence criteria were verified using the Heidelberger and Welch [18] and Geweke [19] criteria, both available in the package “*boa—Bayesian Output Analysis*” [20] of the R software [21].

### 2.4. Estimation of Heritabilities and EBVs over Time

Heritabilities over time for each scenario/sub-scenario in each replicate were calculated as:

(2) $${\hat{h}}_{j}^{2}=\frac{{\hat{\sigma }}_{a_{j}}^{2}}{{\hat{\sigma }}_{a_{j}}^{2}+{\hat{\sigma }}_{q_{j}}^{2}+{\hat{\sigma }}_{p_{j}}^{2}+{\hat{\sigma }}_{e}^{2}},$$

in which ${\hat{h}}_{j}^{2}$ is the heritability estimated for the age j (j = 2, 3, …, 15), ${\hat{\sigma }}_{a_{j}}^{2},{\hat{\sigma }}_{q_{j}}^{2},{\;\mathrm{and}\;\hat{\sigma }}_{p_{j}}^{2}$ are the additive genetic, herd-year-season, and permanent environmental variances estimated for the age j, respectively, and ${\hat{\sigma }}_{e}^{2}$ is the residual variance. The additive genetic, herd-year-season, and permanent environmental variances were obtained from the jth diagonal of the respective covariance matrices for the ages (i.e., ∑, φ, and θ, respectively). The matrices ∑**,** φ, and θ were calculated using the posterior mean of the variance components estimated for the random regression coefficients of these effects, i.e.,:

(3) $$\sum =TG_{0}T\prime ,\;\varphi =TR_{q}T\prime ,\;\mathrm{and}\;\theta =TR_{p}T\prime ,$$

where **T** is a matrix of independent covariates including all ages associated with the Legendre orthogonal polynomial, and $G_{0},\;R_{q},\;\mathrm{and}\;R_{p}$ are the previously mentioned additive genetic, herd-year-season, and permanent environmental variance components matrices for the random regression coefficients, respectively. The heritability estimates reported for each scenario/sub-scenario are the averages (and SE) obtained from three replicates.

The EBVs for all different ages of the animal i, for each scenario/sub-scenario in each replicate, were obtained as:

(4) $$EBV_{i}=T{\hat{a}}_{i},$$

in which $EBV_{i}$ is the vector of EBVs for animal i that includes all analyzed ages, ${\hat{a}}_{i}$ is the vector of breeding values for the regression coefficients of animal i, and **T** is the previously mentioned matrix of independent covariates associated with the Legendre polynomial.

### 2.5. Proportion of Commonly Selected Animals, EBV Correlation, and Computational Efficiency

The proportion of commonly selected animals between COM and the sub-scenarios of CEN, or COM and the sub-scenarios of PEN, was calculated for the top 1% and 10% animals of each age. Similarly, the EBV correlation was calculated as the Pearson correlation coefficient between EBVs predicted using COM and the sub-scenarios of CEN (or PEN), but considering all animals and ages together. Additionally, EBV correlations for the age of four years were calculated using either animals with or without censored/penalized records. Proportions and correlations shown in this study are the averages (and SD) obtained from the three replicates.

All analyses were performed using a Unix server available at the American Angus Association [Intel(R) Xeon(R) CPU E5-2697 v4 @ 2.30 GHz](Intel Corporation, Santa Clara, CA, USA), which contains 72 CPUs and considers up to two threads per core. The total computing time for each replicate of each scenario/sub-scenario was estimated as the amount of CPU time spent in user-mode code plus the amount of CPU time spent in the kernel [22] needed to complete the 150,000 Bayesian iterations. The average (SD) computing time calculated between all three replicates is presented in this study.

### 2.6. Statistical Significance

Statistically significant (*p*-value < 0.05) differences between scenarios, for each longevity indicator, were accessed using paired *t*-tests [23].

## 3. Results

Similar pattern of results was observed using both designs (i.e., always including 55,000 cows with uncensored data; or always including a total of 100,000 cows in the analyses). Thus, only results based on the second design will be presented. Results obtained when using the first design are included in the Supplementary Material and mentioned in the text for comparison purposes, when appropriate.

### 3.1. Heritabilities

**Figure 2:**
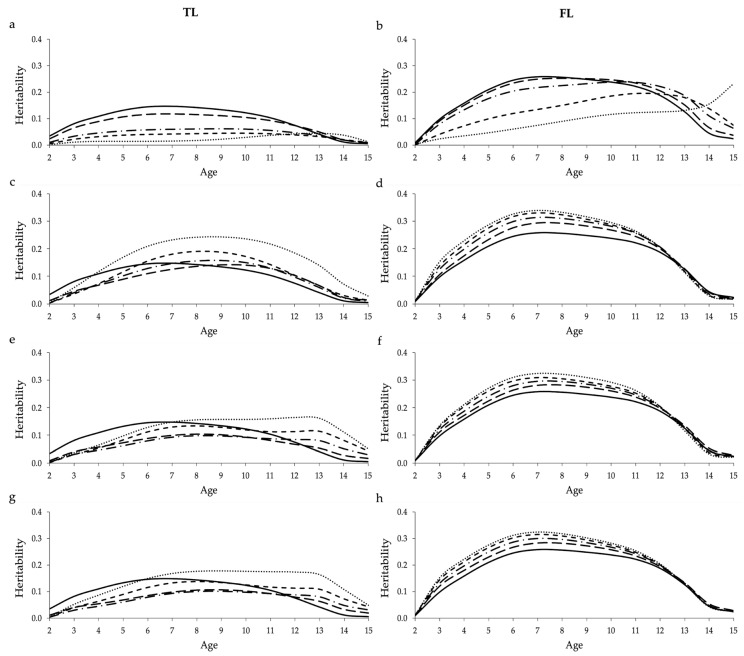
Heritabilities estimated for traditional (TL; **a**,**c**,**e**,**g**) and functional (FL; **b**,**d**,**f**,**h**) longevity across the different ages using 0% (continuous; 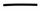), 20% (long dash; 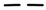), 40% (long dash dot; 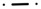), 60% (short dash; 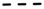), and 80% (round dot; 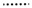) censored data (**a**,**b**); penalized data assuming that all cows with censored records were culled one year after their last reported calving (**c**,**d**); penalized data assuming that only cows with censored records older than nine years were culled one year after their last reported calving (**e**,**f**); and penalized data assuming that cows with censored records older than nine years were culled one year after their last reported calving, while cows younger than nine years were culled two years after their last reported calving (**g**,**h**). Heritabilities shown here are average heritabilities obtained from three replicates. The SE ranged from 0.01 to 0.03.

Heritabilities estimated for TL and FL across the different ages using COM and the sub-scenarios of CEN and PEN are shown in Figure 2. Heritabilities estimated based on the design that always included 55,000 cows with uncensored data are shown in Supplementary Figure S3.

Up to about 12 years old, increasing the proportion of censored data decreased the heritability estimates for both longevity indicators (Figure 2a,b). An overestimation of heritabilities was observed when including censored records at high ages (>12 years), especially for FL (Figure 2b). However, the overall impact of censored data in the heritability estimates was greater for TL than FL. For instance, heritabilities estimated for TL, from 2 to 12 years, reduced on average by 17.13% from COM to CEN20, 55.15% from COM to CEN40, 68.21% from COM to CEN60, and 81.01% from COM to CEN80. For FL, heritabilities reduced on average by 4.06%, 13.27%, 41.54%, and 62.78% over the same mentioned scenarios. Heritabilities estimated using the design that always included 55,000 cows with uncensored data followed a similar pattern than the current design. However, a smaller decrease/increase in the heritability estimates was observed (Supplementary Figure S3).

A similar pattern of heritability was observed among the different sub-scenarios of PEN, within each longevity indicator (Figure 2c–h). In this context, heritabilities estimated for TL tended to be sub-estimated at ages lower than about nine years, and overestimated at greater ages (Figure 2c,e,g). Heritabilities estimated for FL tended to be overestimated until 12 years old, and they were close to the heritabilities estimated using COM after that (Figure 2d,f,h). For both TL and FL, increasing the amount of penalized data emphasized the behavior observed for the heritability pattern (i.e., heritabilities were more under or overestimated when increasing the amount of penalized data). Compared to the use of censored data, the penalty methods tended to yield heritabilities closer to COM, especially for FL (Figure 2b,d,f,h). Heritabilities estimated using the second and third penalization criteria were more similar among them than with the first penalization criterion using both longevity indicators (Figure 2c–h). For the design with 55,000 cows with uncensored data, all sub-scenarios of PEN yielded heritabilities closer to COM compared to the 100,000 cows design (Figure S3, Supplementary Material).

Significant statistical differences were observed between average heritabilities estimated using different proportions of censored data for TL. On the other hand, no statistical differences were observed between heritabilities estimated for FL using COM (i.e., 0%) and smaller proportions of censored data (i.e., 20% and 40%). Using penalized data significantly increased the average heritabilities for both longevity indicators. Consequently, no statistical differences were observed between average heritabilities estimated using COM and the majority of sub-scenarios of PEN, for both longevity indicators. The average heritability estimates and significance of the associated differences in estimates across scenarios within each longevity indicator (TL or FL) are shown in Table S1 (Supplementary Material). No significant statistical differences were observed for the design that always included 55,000 cows with uncensored data (results not shown).

### 3.2. Proportion of Commonly Selected Animals

**Figure 3:**
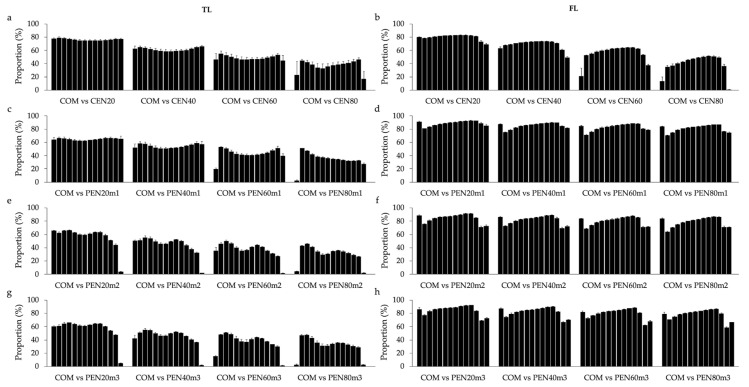
Average proportion (and SE) of the top 10% animals commonly selected for each age (black bars; from 2 to 15 years old) across the different scenarios, using the traditional (TL; **a**,**c**,**e**,**g**) and functional (FL; **b**,**d**,**f**,**h**) longevity indicators. The contrasted scenarios are: complete (COM; without censored/penalized records); randomly censoring different proportions of data (CEN20, CEN40, CEN60, and CEN80 for 20%, 40%, 60% and 80% censored, respectively; (**a**,**b**); penalizing data assuming that all cows with censored records were culled one year after their last reported calving (PEN20m1, PEN40m1, PEN60m1, and PEN80m1 for 20%, 40%, 60% and 80% penalized, respectively; (**c**,**d**); penalizing data assuming that only cows with censored records older than nine years were culled one year after their last reported calving (PEN20m2, PEN40m2, PEN60m2, and PEN80m2 for 20%, 40%, 60% and 80% penalized, respectively; (**e**,**f**); and penalizing data assuming that cows with censored records older than nine years were culled one year after their last reported calving, while cows younger than nine years were culled two years after their last reported calving (PEN20m3, PEN40m3, PEN60m3, and PEN80m3 for 20%, 40%, 60% and 80% penalized, respectively; (**g**,**h**). The averages (and SE) shown here were obtained from three replicates.

The average proportions (and SE) of top 10% animals selected in common for each age between COM and the different sub-scenarios of CEN and PEN are shown in Figure 3. The average proportions (and SE) of the top 1% animals commonly selected are shown in Supplementary Figure S4. Corresponding results obtained based on the design using 55,000 cows with uncensored data are shown in Supplementary Figures S5 and S6, respectively.

In summary, the proportion of animals commonly selected between COM and CEN reduced when the proportion of cows with censored data increased (Figure 3a,b). Including penalized data while analyzing TL tended to increase re-ranking of animals between COM and PEN compared to COM and CEN (Figure 3a,c,e,g). On the other hand, including penalized data for FL increased the proportion of commonly selected animals, specially under sub-scenarios with great amount of censored/penalized data (i.e., >40%; Figure 3b,d,f,h). Higher variability in the proportion of animals commonly selected across ages was observed for TL compared to FL, for all sub-scenarios of PEN (Figure 3c–h). In addition, higher variability between ages tended to be observed when considering the top 1% animals selected in common (Supplementary Figure S4) instead of the top 10%. Similar patterns were observed for the design with 55,000 cows with uncensored data (Figures S5 and S6, Supplementary Material).

The average proportion of the top 10% animals selected in common between COM and all sub-scenarios of CEN and PEN, considering all ages, are shown in Table S2 (Supplementary Material). In general, similar average proportions of animals selected in common were found for TL and FL when using censored data. However, when using penalized data the proportions were higher for FL than for TL, in all scenarios analyzed. For TL, the highest proportion of animals commonly selected was found between COM and CEN20 (76.5%), while the lowest proportions (about 35.5% animals in common) were found between COM and the scenarios with a great amount of censored (CEN80) or penalized (PEN80m1, PEN60m2, PEN80m2, PEN60m3, and PEN80m3) data. For FL, the average proportions were over 78% when using CEN20 and all scenarios using penalized data. The lowest proportion of commonly selected animals for FL was found between COM and CEN80 (39.4%). Similar proportions were observed for the design that always included 55,000 cows with uncensored data (results not shown).

### 3.3. EBV Correlation

**Figure 4:**
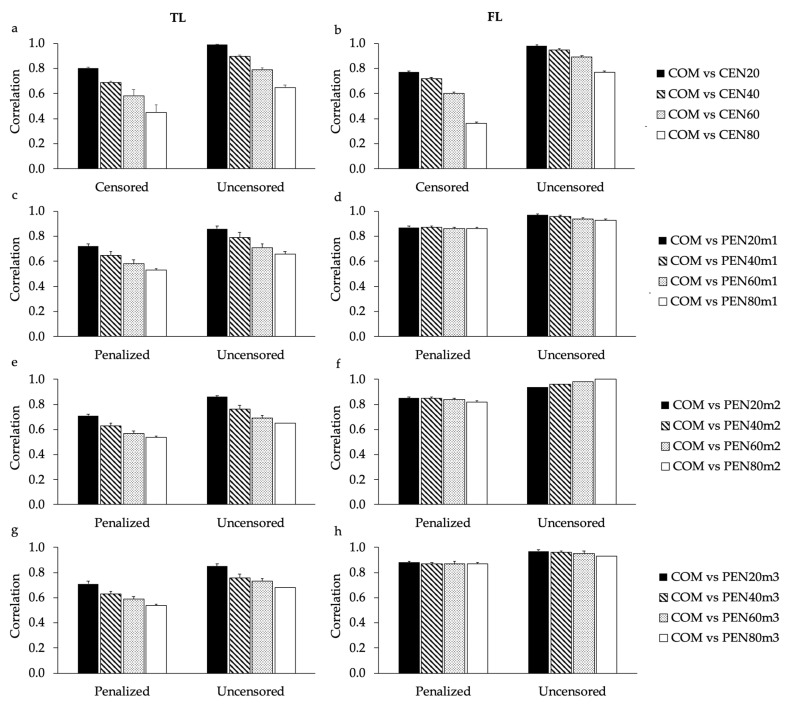
Average correlation (and SE) estimated between breeding values predicted for traditional (TL; **a**,**c**,**e**,**g**) and functional (FL; **b**,**d**,**f**,**h**) longevity, at the age of four years, using different scenarios. Contrasted scenarios are: complete (COM; without censored/penalized records); randomly censoring different proportions of data (CEN20, CEN40, CEN60, and CEN80 for 20%, 40%, 60% and 80% censored, respectively; (**a**,**b**); penalizing data assuming that all cows with censored records were culled one year after their last reported calving (PEN20m1, PEN40m1, PEN60m1, and PEN80m1 for 20%, 40%, 60% and 80% penalized, respectively; (**c**,**d**); penalizing data assuming that only cows with censored records older than nine years were culled one year after their last reported calving (PEN20m2, PEN40m2, PEN60m2, and PEN80m2 for 20%, 40%, 60% and 80% penalized, respectively; (**e**,**f**); and penalizing data assuming that cows with censored records older than nine years were culled one year after their last reported calving, while cows younger than nine years were culled two years after their last reported calving (PEN20m3, PEN40m3, PEN60m3, and PEN80m3 for 20%, 40%, 60% and 80% penalized, respectively; (**g**,**h**). Correlations were estimated considering only cows with or without censored/penalized records (Censored/Penalized and Uncensored, respectively). Averages (and SE) shown here were obtained from three replicates.

The average correlations estimated between EBV predicted using COM and all sub-scenarios of CEN and PEN, when considering all ages together, are shown in Table 1. Similar to the results observed for the proportion of commonly selected animals, high EBV correlations were estimated between COM and CEN20 (for both TL and FL), and COM and all sub-scenarios of PEN (for FL). The inclusion of penalized data decreased the EBV correlations for TL when compared to CEN20 (Table 1). Similar EBV correlations were calculated for the sub-scenarios of PEN using the design that always included 55,000 cows with uncensored data (Table S3, Supplementary Material). However, EBV correlations estimated between COM and the sub-scenarios of CEN tended to decrease less with the increase in the proportion of censored data for the design using the 55,000 cows compared to the design using 100,000 cows (Table 1 and Supplementary Table S3). The impact of the different scenarios in the EBV correlation when considering only cows that had or not censored/penalized records was calculated at the age of four years (Figure 4). The average EBV correlation estimated for cows that had or not censored/penalized records, calculated using the design of 55,000 cows, are shown in Figure S6 (Supplementary Material).

In all scenarios evaluated, the EBV correlations estimated at four years were lower when considering only cows that had their culling information censored or penalized compared to cows that had uncensored records (Figure 4). For both TL and FL, the EBV correlation decreased as the proportion of censored data increased (Figure 4a,b). Especially for TL, the EBV correlations tended to be lower for PEN than for CEN (Figure 4a,c,e,g). However, for FL the EBV correlations estimated when using PEN were higher than the EBV correlations estimated using CEN (Figure 4b,d,f,h). It is worth noting that the EBV correlations estimated using the design that always included 55,000 cows with uncensored data were similar to the ones previously described for cows that had or not their culling information penalized (i.e., for all sub-scenarios of PEN). However, the EBV correlations estimated for censored and uncensored cows, using the design that always included 55,000 cows, tended to decrease less with the increase of censored data compared to the 100,000 cows design (Figure 4 and Figure S7). Similar EBV correlations were estimated using the different penalty methods in both designs (Figure 4c–h and Figure S7c–h).

### 3.4. Computational Efficiency

The averages CPU time used to complete the 150,000 Bayesian iterations using COM and the sub-scenarios of CEN and PEN are shown in Table 2. In summary, analysis using COM and CEN tended to take longer than analyses using PEN. In addition, increasing the amount of censored records also tended to increase the computational time spent in the analyses. In this context, analysis using only penalized data (i.e., PENxm1 and PENxm3) tended to be faster than analyses including censored data, especially for FL. Similar results were observed for the design that always included 55,000 cows with uncensored data (results not shown).

## 4. Discussion

Beef cattle longevity is strongly related to the overall farm profitability [5]. Due to its importance, several longevity indicators have been proposed over the years [24,25,26,27]. Recently, Oliveira et al. [6] contrasted the use of different longevity indicators to genetically evaluate longevity in North American Angus cattle, but without accounting for the presence of censored records in the evaluations. Understanding the impact of censored records and how to account for them in the genetic evaluations of longevity were the main motivations for this study. Therefore, three criteria were proposed for the penalty methods, which differed mainly regarding to the threshold used to create the penalized data. In summary, the first criterion assumed the culling date for each cow as one year after its last reported calving, which can be very stringent for young cows. Thus, the second and third criteria used tried to reduce the possible disadvantage of the first criterion for the young cows, in order to avoid bias and reduction of genetic gain.

Genetic evaluations of longevity usually rely on animal culling records reported by the farmers. However, probably because longevity has not been officially evaluated in North American Angus cattle [28,29], a great proportion of cows (~62%) present in the pedigree do not have culling records included in the original dataset [6]. This proportion is higher than the ones previously reported in the literature for other beef cattle breeds. For instance, Forabosco et al. [30] reported that about 14% of cows had censored data in longevity analyses of the Chianina beef cattle breed. Brzáková et al. [26] reported that about 37% of cows from several beef cattle breeds raised in Czech Republic had censored data. Thus, in order to mimic different scenarios for beef cattle, the proportions of cows with censored records in the current study were set to 20%, 40%, 60%, and 80%.

Including all animals with censored records in the genetic evaluation can help to increase the accuracy of EBVs for longevity [9,31]. Moreover, the use of more information can also improve the estimation of variance components and genetic parameters [31]. However, the great challenge is to determine how to best include censored records in the evaluations. Methods described in the literature to account for censored data in the genetic evaluation include the use of augmented data (created using penalization criteria [12,32] or replacing censored records by simulated values originated from truncated normal distributions [12,32,33,34]), and the use of missing values [12,27,32]. Either way, directly removing censored data from the analysis has not been recommended [9,32].

Merely excluding censored data from the genetic evaluation of fertility traits was shown to favor sires that have a greater proportion of daughters with censored records, because the average value of their censored records is usually greater than the population mean calculated for the trait [9]. In this context, Donoghue et al. [32], evaluating different methods to handle censored records for fertility traits in Australian Angus cattle, concluded that predictions made using augmented data generated by the penalty method and by the simulated values originated from predictive truncated normal distributions were very similar. However, the authors comment that both methods perform better than excluding the censored records from the genetic evaluation. Thus, the use of statistical models that enable analyzing binary traits over time is proposed to keep censored records (assumed as missing or augmented records) in the analysis without mischaracterizing the trait [12,32,35]. In this context, using RRM seems an optimal choice, because it combines the information of all repeated records without requiring all animals having records at all time-points [13,14]. Specifically, for longevity related traits, the use of RRM allows to include animals culled at different ages in the same genetic evaluation. This feature excludes the need to evaluate longevity at specific time-points (e.g., six years; [36]), and assures that EBVs are predicted for all animals within the range of all evaluated ages [13,14].

Using missing values to account for censored data in the RRM significantly decreased the average heritabilities estimated for TL and FL across ages, especially when high proportions of censored data where included in the analysis using the design based on 100,000 cows (Table S1 and Figure 2). Moreover, a trend of decrease was observed while analyzing the heritabilities estimated over the ages, using both data designs (Figure 2 and Figure S3). The lower heritability estimates observed when increasing the proportion of censored data is mainly due to the reduction in the additive genetic variance (Supplementary Figure S8). These results suggest that including high proportions of censored data may hamper the genetic progress for longevity in North American Angus cattle. However, when small proportions of censored data (20%) were used, the estimated pattern of heritabilities was similar to COM. These results corroborate the ones presented by Donoghue et al. [35], who reported that genetic parameters estimated using 12% and 20% censored data (treated as missing) were similar to the true values generated in the simulation. On the other hand, Forabosco et al. [30] reported lower heritability estimates when only uncensored records were used. The different findings of the current study might be justified by the different statistical models used (survival versus RRM). A trend of decrease in the sire variances similar to the one observed for the additive genetic variances estimated in this study was reported by Guo et al. [37], while studying the influence of censored data on the genetic parameters estimated for performance and prolificacy traits in swine. Among the longevity indicators tested, heritabilities estimated for FL seem to be less impacted by the amount of censored data compared to TL, probably due to the fact that calving information is also included in FL.

Heritabilities estimated for FL in the different sub-scenarios of PEN, based on the design that always included 100,000 cows, tended to be overestimated (Figure 2 and Supplementary Table S1). This suggests that the use of penalty method can likely contribute to increasing the genetic variability when the proportion of uncensored cows is reduced in the analysis (Supplementary Figure S8). However, when the proportion of uncensored cows remains constant (design with 55,000 cows), genetic parameters predicted using PEN are very similar to the real ones (COM). For both designs, similar genetic parameters were estimated using the three penalty criteria. These findings indicate that the criteria proposed in this study for the penalty method might be useful to estimate genetic parameters for FL. On the other hand, the criteria used here do not seem adequate for TL, as heritabilities estimated were mostly biased. A possible explanation for this is that TL (as defined in this study) does not take into account calving information, and the three penalization criteria proposed in this study are based on the information of missing calvings. This result reinforces the importance of using appropriate penalization criteria for the analyzed trait. Donoghue et al. [32], using data from fertility traits of Australian Angus cattle, found that different methods used to generate augmented data had small impact in the estimation of genetic parameters and additive genetic variance. Moreover, the authors reported that the penalty method does not significantly overestimate the genetic parameters compared to censored records [32].

Similar pattern of results was found for the proportion of animals commonly selected using both designs (i.e., always including 55,000 cows with uncensored data, or always including a total of 100,000 cows in the analysis). For both longevity indicators the proportion of animals commonly selected between COM and CEN reduced when the proportion of cows with censored data increased (Figure 3 and Supplementary Table S2). These findings corroborate the idea that including great proportions of censored data in the genetic analysis of longevity can have a negative impact in the selection process. Moreover, the proportion of animals commonly selected between COM and PEN support the hypothesis that the penalty methods proposed in this study are appropriate to analyze FL, but not for TL. In this context, the high average proportions (over 78%) of animals selected in common between COM and PEN for FL indicate that similar selection decisions are made when using COM, CEN20, and all sub-scenarios of PEN. However, some re-ranking of animals is expected. These results seem to corroborate in part with the ones reported by Donoghue et al. [32,35], as no major re-ranking of sires were reported by the authors when investigating censored data for days to calving in simulated and real beef cattle data, respectively.

Correlations of EBVs estimated in this study considering all animals together (with censored and uncensored records; Table 1), support the results found for the high proportion of commonly selected animals between COM and CEN20 (for both TL and FL), and COM and all sub-scenarios of PEN (for FL). In order to investigate the impact of censored records in the EBV correlations using only cows that had or not censored/penalized records, the age of four years was used (Figure 4 and Supplementary Figure S6). Breeding values predicted for the age of four years were previously recommended for genetic selection of longevity in North American Angus cattle, as they were expected to result in greater selection responses using the longevity indicators analyzed in this study [6]. As expected, EBV correlations estimated for cows that had their culling information censored tended to be lower than EBV correlations estimated for cows that had only uncensored records. However, it is worth noting that the inclusion of penalized data for cows with previously censored records impacts the EBVs predicted for uncensored animals (Figure 4 and Figure S7). This impact is due to the fact that all related animals contribute to the EBVs predicted using the BLUP method [38], as they are mostly connected through the **A** matrix. Moreover, the impact of the amount of censored records in the EBV correlations calculated using the design based on the 55,000 cows with uncensored records was lower than the impact observed using the design based on 100,000 cows (Figure 4 and Supplementary Figure S7). These findings might be related to the fact that for the design including the 55,000 cows, the number of informative records were not reduced among sub-scenarios. Setiaji et al. [39], analyzing different penalty methods to access interval from the first to the last successful insemination in Japanese Black heifers, concluded that EBV correlations decrease at higher levels of censored (missing) records. Using only uncensored data and the conventional linear model was also the most recommended method for the genetic evaluation of age at first calving in Brahman cattle [11]. However, the authors pointed out that similar EBV correlations were observed using either uncensored or penalized data, which suggests that there was not relevant re-ranking of animals when censored records were used [11]. Similarly, Costa et al. [40] also found that the linear-threshold model without censored data showed the best predictive ability (computed based on the EBV correlation estimated between the complete and reduced datasets) for the genetic evaluation of both age at first calving and stayability in Nellore cattle. On the other hand, Veerkamp et al. [41] showed that RRM were relatively robust to censoring in genetic evaluations of survival in dairy cattle, as similar EBV predictions were made based on uncensored and censored data.

Even though the scheme of the designs, scenarios, and sub-scenarios evaluated in the study tried to mimic real data, it is important to highlight here the need to re-estimate variance components using the complete dataset available for North American Angus. In addition, further studies comparing the use of different approaches (e.g., multiple-trait model without censored/penalized records) are required to simplify the pipeline for the official genetic evaluations. In this regard, it is worth to note that including animals with censored records in the genetic analysis of longevity might not be relevant if genomic information is included, as high prediction accuracies are already expected for young animals using genomic selection [42,43,44]. Thus, especially for selection of candidates at young ages (e.g., when animals are still alive), the increase in accuracy observed in genomic evaluations might enable considerably a reduction in the generation interval. Reduction in the generation interval is highly recommended when analyzing longevity because the productive life of beef cows can be relatively long (i.e., they can easily exceed five years [30]). Moreover, reducing the generation interval can contribute to significantly increase the genetic gain per unit time. In this regard, Ramos et al. [25] suggested that genomic information should always be included in the evaluations performed for longevity-related traits, especially when they are evaluated at early ages. However, further studies are needed to evaluate the performance of genomic selection for longevity-related traits in North American Angus cattle.

In order to evaluate the feasibility of the use of censored or penalized data in the genetic evaluation of longevity using RRM, the computational efficiency was also investigated (Table 2). In summary, for all scenarios/sub-scenarios analyzed in this study, more than eight days were needed to complete the 150,000 iterations. This indicates that further improvements to reduce computational time are required to allow genetic evaluations to be run in a reasonable time frame. However, despite its high computational time, using RRM seems to be suitable to analyze longevity in North American Angus cattle considering COM, CEN, and PEN. Concerns regarding computational time are especially relevant for genomic evaluations, as a recent study showed that incorporating genomic information into genetic evaluations based on RRM increased the computational demand for the evaluation of several traits [22].

## 5. Conclusions

High proportions (>20%) of censored data have a negative impact on the genetic evaluation of longevity. The penalization criteria proposed in this study are not recommended when analyzing traditional longevity, however, they are useful for genetic evaluations of functional longevity. In this context, the three penalization criteria will have similar impact in the breeding program, as small differences were observed in the genetic parameters, proportion of animals commonly selected, and breeding values estimated. Improvements to reduce computational time are required for routine genetic and genomic evaluation for longevity in North American Angus cattle.

## Figures and Tables

**Table 1:** Average (SD) correlation estimated between breeding values predicted for traditional (TL) and functional (FL) longevity using the complete data and all other scenarios, considering all ages combined (i.e., from 2 to 15 years old).

Longevity Indicator	^1^ Scenario	Proportion of Censored/Penalized Data			
		20%	40%	60%	80%
TL	CEN	0.90 (0.03) a	0.80 (0.04) b	0.68 (0.05) d	0.51 (0.11) f,g,h
	PENxm1	0.80 (0.03) b	0.69 (0.05) d	0.55 (0.11) e,f,g	0.43 (0.22) h,i
	PENxm2	0.73 (0.07) c,d	0.58 (0.10) e,f	0.47 (0.11) h,i	0.39 (0.18) i
	PENxm3	0.74 (0.06) c	0.59 (0.08) e	0.49 (0.11) g,h	0.41 (0.24) h,i
FL	CEN	0.91 (0.05) d	0.84 (0.07) e	0.73 (0.13) f	0.49 (0.24) g
	PENxm1	0.97 (0.03) a	0.97 (0.03) a	0.96 (0.03) a,b	0.95 (0.03) a,b,c
	PENxm2	0.93 (0.03) c,d	0.93 (0.04) c,d	0.92 (0.04) c,d	0.90 (0.05) d
	PENxm3	0.94 (0.03) b,c	0.94 (0.03) b,c	0.93 (0.03) c,d	0.92 (0.04) c,d

^1^ Scenarios included different proportions of censored data (CEN); penalized data assuming that all cows with censored records were culled one year after their last reported calving (PENxm1); penalizing data assuming that only cows with censored records older than nine years were culled one year after their last reported calving (PENxm2); and penalizing data assuming that cows with censored records older than nine years were culled one year after their last reported calving, while cows younger than nine years were culled two years after their last reported calving (PENxm3). Different letters, within each longevity indicator, show significant difference (*p*-value < 0.05) between values. Averages (and SD) shown here were obtained across years and three replicates.

**Table 2:** Average (SD) central processing unit (CPU) time (in days) used to complete 150,000 Bayesian iterations in the genetic evaluation of traditional (TL) and functional (FL) longevity using different scenarios.

Longevity Indicator	^1^ Scenario	Proportion of Censored/Penalized Data				
		0%	20%	40%	60%	80%
TL	CEN	10.32 (0.26) c	10.49 (0.95) c	10.92 (0.77) b,c	11.56 (1.15) a,b	11.75 (0.88) a
	PENxm1	10.32 (0.26) c	9.12 (0.03) e	9.30 (0.07) d	9.43 (0.10) d	9.61 (0.07) d
	PENxm2	10.32 (0.26) c	9.60 (0.55) d	9.67 (0.72) d	9.61 (0.75) d	9.59 (0.81) d
	PENxm3	10.32 (0.26) c	9.38 (0.03) d	9.59 (0.02) d	9.74 (0.05) d	9.74 (0.01) d
FL	CEN	9.74 (1.09) b	10.33 (1.17) a	10.67 (1.12) a	11.30 (1.52) a	11.32 (1.48) a
	PENxm1	9.74 (1.09) b	8.26 (0.06) c	8.43 (0.04) c	8.22 (0.02) c	8.20 (0.03) c
	PENxm2	9.74 (1.09) b	9.65 (0.19) b	9.71 (0.35) b	9.77 (0.49) b	9.68 (0.67) b
	PENxm3	9.74 (1.09) b	8.55 (0.03) c	8.49 (0.02) c	8.48 (0.03) c	8.28 (0.03) c

^1^ Scenarios included different proportions of censored data (CEN); penalized data assuming that all cows with censored records were culled one year after their last reported calving (PENxm1); penalizing data assuming that only cows with censored records older than nine years were culled one year after their last reported calving (PENxm2); and penalizing data assuming that cows with censored records older than nine years were culled one year after their last reported calving, while cows younger than nine years were culled two years after their last reported calving (PENxm3). Different letters, within each longevity indicator, show significant difference (*p*-value < 0.05) between values. Averages (and SD) shown here were obtained using the three replicates.

## Data Availability

All relevant information supporting the results of this study are included within the paper and its supplementary material. The raw data cannot be made available, as it is property of the North American Angus producers.

## Supplementary Materials

The following are available online at https://www.mdpi.com/2076-2615/11/3/800/s1. Figure S1: Distribution of the number of cows culled per age (cumulative). Figure S2: Proportion of reappearance in the dataset after one missing calving. Figure S3: Heritabilities estimated for traditional and functional longevity over the different ages using 55,000 cows with uncensored records. Figure S4: Average proportion (and SE) of top 1% animals selected in common for each age (from 2 to 15 years) between the different scenarios, using the traditional and functional longevity. Figure S5: Average proportion (and SE) of top 1% animals selected in common for each age (from 2 to 15 years) between the different scenarios, using the design based on 55,000 cows with uncensored records for traditional and functional longevity. Figure S6: Average proportion (and SE) of top 10% animals selected in common for each age (from 2 to 15 years) between the different scenarios, using the design based on 55,000 cows with uncensored records for traditional and functional longevity. Figure S7: Average correlation (and SE) estimated between breeding values predicted using the design of 55,000 cows with uncensored records for traditional and functional longevity, at the age of four years, using the different scenarios. Figure S8: Additive genetic variance estimated for traditional and functional longevity over the different ages. Table S1: Average heritability (SD) estimates for traditional (TL) and functional (FL) longevity considering all ages (i.e., from 2 to 15 years old) for each scenario. Table S2: Average (SD) proportion of top 10% animals selected in common between the complete data and all other scenarios for traditional (TL) and functional (FL) longevity, considering all ages (i.e., from 2 to 15 years old). Table S3: Average (SD) correlation estimated between breeding values predicted for traditional and functional longevity using the complete data and all other scenarios, considering all ages (i.e., from 2 to 15 years) together and the design of 55,000 cows with uncensored records.
