# Supplementary material for: Impact of Censored or Penalized Data in the Genetic Evaluation of Two Longevity Indicator Traits Using Random Regression Models in North American Angus Cattle
Source: Animals (Basel). 2021 Mar 12;11(3):800. doi: 10.3390/ani11030800 (PMC8001693; doi:10.3390/ani11030800)
Supplement: Supplementary file 1 [file animals-11-00800-s001.zip › SupplementaryMaterial_ok1.docx]

**SUPPLEMENTARY MATERIAL**

**Figure S1.** Distribution of the number of cows culled per age (cumulative).

**Figure S2.** Proportion of reappearance in the dataset after one missing calving. N: number of cows.

**Figure S3.** Heritabilities estimated for traditional (TL; a, c, e, and g) and functional (FL; b, d, f, and h) longevity over the different ages using 55,000 cows with uncensored records plus 0% (continuous;), 20% (long dash;), 40% (long dash dot;), 60% (short dash;), and 80% (round dot;) cows with censored data (a, b); penalized data assuming that all cows with censored records were culled one year after their last reported calving (c, d); penalized data assuming that only cows with censored records older than nine years were culled one year after their last reported calving (e, f); and penalized data assuming that cows with censored records older than nine years were culled one year after their last reported calving, while cows younger than nine years were culled two years after their last reported calving (g, h). Heritabilities shown here are average heritabilities obtained from the three replicates. The SE ranged from 0.01 to 0.02.

**Figure S4.** Average proportion (and SE) of top 1% animals selected in common for each age (black bars; from 2 to 15 years) between the different scenarios, using the traditional (TL; a, c, e, and g) and functional (FL; b, d, f, and h) longevity. Contrasted scenarios are: complete (COM; without censored/penalized records); randomly censoring different proportions of data (CEN20, CEN40, CEN60, and CEN80 for 20%, 40%, 60% and 80% censored, respectively; a and b); penalizing data assuming that all cows with censored records were culled one year after their last reported calving (PEN20m1, PEN40m1, PEN60m1, and PEN80m1 for 20%, 40%, 60% and 80% penalized, respectively; c and d); penalizing data assuming that only cows with censored records older than nine years were culled one year after their last reported calving (PEN20m2, PEN40m2, PEN60m2, and PEN80m2 for 20%, 40%, 60% and 80% penalized, respectively; e and f); and penalizing data assuming that cows with censored records older than nine years were culled one year after their last reported calving, while cows younger than nine years were culled two years after their last reported calving (PEN20m3, PEN40m3, PEN60m3, and PEN80m3 for 20%, 40%, 60% and 80% penalized, respectively; g and h). Averages (and SE) shown here were obtained from the three replicates.

**Figure S5.** Average proportion (and SE) of top 1% animals selected in common for each age (black bars; from 2 to 15 years) between the different scenarios, using the design based on 55,000 cows with uncensored records for traditional (TL; a, c, e, and g) and functional (FL; b, d, f, and h) longevity. Contrasted scenarios are: complete (COM; without censored/penalized records); randomly censoring different proportions of data (CEN20, CEN40, CEN60, and CEN80 for 20%, 40%, 60% and 80% censored, respectively; a and b); penalizing data assuming that all cows with censored records were culled one year after their last reported calving (PEN20m1, PEN40m1, PEN60m1, and PEN80m1 for 20%, 40%, 60% and 80% penalized, respectively; c and d); penalizing data assuming that only cows with censored records older than nine years were culled one year after their last reported calving (PEN20m2, PEN40m2, PEN60m2, and PEN80m2 for 20%, 40%, 60% and 80% penalized, respectively; e and f); and penalizing data assuming that cows with censored records older than nine years were culled one year after their last reported calving, while cows younger than nine years were culled two years after their last reported calving (PEN20m3, PEN40m3, PEN60m3, and PEN80m3 for 20%, 40%, 60% and 80% penalized, respectively; g and h). Averages (and SE) shown here were obtained from the three replicates.

**Figure S6.** Average proportion (and SE) of top 10% animals selected in common for each age (black bars; from 2 to 15 years) between the different scenarios, using the design based on 55,000 cows with uncensored records for traditional (TL; a, c, e, and g) and functional (FL; b, d, f, and h) longevity. Contrasted scenarios are: complete (COM; without censored/penalized records); randomly censoring different proportions of data (CEN20, CEN40, CEN60, and CEN80 for 20%, 40%, 60% and 80% censored, respectively; a and b); penalizing data assuming that all cows with censored records were culled one year after their last reported calving (PEN20m1, PEN40m1, PEN60m1, and PEN80m1 for 20%, 40%, 60% and 80% penalized, respectively; c and d); penalizing data assuming that only cows with censored records older than nine years were culled one year after their last reported calving (PEN20m2, PEN40m2, PEN60m2, and PEN80m2 for 20%, 40%, 60% and 80% penalized, respectively; e and f); and penalizing data assuming that cows with censored records older than nine years were culled one year after their last reported calving, while cows younger than nine years were culled two years after their last reported calving (PEN20m3, PEN40m3, PEN60m3, and PEN80m3 for 20%, 40%, 60% and 80% penalized, respectively; g and h). Averages (and SE) shown here were obtained from the three replicates.

**Figure S7.** Average correlation (and SE) estimated between breeding values predicted using the design of 55,000 cows with uncensored records for traditional (TL; a, c, e, and g) and functional (FL; b, d, f, and h) longevity, at the age of four years, using the different scenarios. Contrasted cenarios are: complete (COM; without censored/penalized records); randomly censoring different proportions of data (CEN20, CEN40, CEN60, and CEN80 for 20%, 40%, 60% and 80% censored, respectively; a and b); penalyzing data assuming that all cows with censored records were culled one year after their last reported calving (PEN20m1, PEN40m1, PEN60m1, and PEN80m1 for 20%, 40%, 60% and 80% penalized, respectively; c and d); penalizing data assuming that only cows with censored records older than nine years were culled one year after their last reported calving (PEN20m2, PEN40m2, PEN60m2, and PEN80m2 for 20%, 40%, 60% and 80% penalized, respectively; e and f); and penalizing data assuming that cows with censored records older than nine years were culled one year after their last reported calving, while cows younger than nine years were culled two years after their last reported calving (PEN20m3, PEN40m3, PEN60m3, and PEN80m3 for 20%, 40%, 60% and 80% penalized, respectively; g and h). Correlations were estimated considering only cows with or without censored/penalized records (Censored/Penalized and Uncensored, respectively). Averages (and SE) shown here were obtained from the three replicates.

**Figure S8.** Additive genetic variances estimated for traditional (TL; a, c, e, and g) and functional (FL; b, d, f, and h) longevity over the different ages using 100,000 cows from which 0% (continuous;), 20% (long dash;), 40% (long dash dot;), 60% (short dash;), and 80% (round dot;) cows had censored data (a, b); penalized data assuming that all cows with censored records were culled one year after their last reported calving (c, d); penalized data assuming that only cows with censored records older than nine years were culled one year after their last reported calving (e, f); and penalized data assuming that cows with censored records older than nine years were culled one year after their last reported calving, while cows younger than nine years were culled two years after their last reported calving (g, h). Variances shown here are averages obtained from the three replicates. The SE ranged from 0.000 to 0.003.

**Table 1.** Average heritabilities (SD) estimated for traditional (TL) and functional (FL) longevity considering all ages (i.e., from 2 to 15 years old), for each scenario.

| **Longevity**  **indicator** | **^1^Scenario** | **Proportion of censored/penalized data** | | | | |
| --- | --- | --- | --- | --- | --- | --- |
|  |  | **0%** | **20%** | **40%** | **60%** | **80%** |
| **TL** | **CEN** | 0.09 (0.05)b,c | 0.05 (0.04)c,d | 0.04 (0.02)d | 0.03 (0.01)d | 0.02 (0.01)e |
|  | **PENxm1** | 0.09 (0.05)b,c | 0.09 (0.05)b,c | 0.09 (0.06)b,c | 0.11 (0.07)a,b | 0.15 (0.08)a |
|  | **PENxm2** | 0.09 (0.05)b,c | 0.06 (0.03)c | 0.07 (0.03)c | 0.09 (0.04)b,c | 0.11 (0.05)a,b |
|  | **PENxm3** | 0.09 (0.05)b,c | 0.07 (0.03)c | 0.07 (0.03)c | 0.09 (0.04)b,c | 0.13 (0.06)a,b |
| **FL** | **CEN** | 0.17 (0.09)a,b | 0.17 (0.09)a,b | 0.17 (0.08)a,b | 0.13 (0.06)b,c | 0.09 (0.06)c |
|  | **PENxm1** | 0.17 (0.09)a,b | 0.19 (0.10)a | 0.20 (0.11)a | 0.20 (0.12)a | 0.21 (0.12)a |
|  | **PENxm2** | 0.17 (0.09)a,b | 0.19 (0.10)a | 0.19 (0.10)a | 0.20 (0.11)a | 0.20 (0.12)a |
|  | **PENxm3** | 0.17 (0.09)a,b | 0.19 (0.10)a | 0.19 (0.10)a | 0.20 (0.11)a | 0.20 (0.11)a |

***^1^***Scenarios included different proportions of censored data (CEN); penalized data assuming that all cows with censored records were culled one year after their last reported calving (PENxm1); penalizing data assuming that only cows with censored records older than nine years were culled one year after their last reported calving (PENxm2); and penalizing data assuming that cows with censored records older than nine years were culled one year after their last reported calving, while cows younger than nine years were culled two years after their last reported calving (PENxm3). Proportion of 0% correspond to the complete data (i.e., 0% censored or penalized data). Different letters, within each longevity indicator, show significant difference (p‐value <0.05) between values. Averages (and SD) showed here were obtained over the years and three replicates. .

**Table S2.** Average (SD) proportion of top 10% animals selected in common between the complete data and all other scenarios, for traditional (TL) and functional (FL) longevity considering all ages (i.e., from 2 to 15 years old).

| **Longevity**  **indicator** | **^1^Scenario** | **Proportion of censored/penalized data** | | | |
| --- | --- | --- | --- | --- | --- |
|  |  | **20%** | **40%** | **60%** | **80%** |
| **TL** | **CEN** | 76.50 (1.50)a | 61.79 (2.50)c | 48.99 (3.17)e | 36.92 (8.17)g |
|  | **PENxm1** | 64.90 (1.60)b | 54.64 (2.92)d | 45.04 (4.52)f | 36.94 (6.60)g |
|  | **PENxm2** | 56.48 (16.3)c,d | 44.49 (13.71)e,f,g | 36.71 (11.81)g | 30.44 (12.72)g |
|  | **PENxm3** | 56.72 (16.43)c,d | 44.79 (13.88)e,f,g | 38.24 (12.55)g | 33.68 (11.12)g |
| **FL** | **CEN** | 80.12 (4.11)c | 68.96 (6.91)d | 55.84 (12.28)e | 39.41 (14.97)f |
|  | **PENxm1** | 88.80 (3.65)a | 85.33 (4.37)b | 82.92 (5.04)b,c | 81.19 (5.14)c |
|  | **PENxm2** | 84.56 (6.65)b,c | 81.86 (6.42)b,c | 80.28 (6.46)c | 78.92 (7.21)c |
|  | **PENxm3** | 84.79 (7.08)b,c | 82.36 (7.22)b,c | 80.00 (7.58)c | 78.26 (8.01)c |

***^1^***Scenarios included different proportions of censored data (CEN); penalized data assuming that all cows with censored records were culled one year after their last reported calving (PENxm1); penalizing data assuming that only cows with censored records older than nine years were culled one year after their last reported calving (PENxm2); and penalizing data assuming that cows with censored records older than nine years were culled one year after their last reported calving, while cows younger than nine years were culled two years after their last reported calving (PENxm3). Different letters, within each longevity indicator, indicate significant difference (p‐value <0.05) between values. Averages (and SD) shown here were obtained across years and three replicates.

**Table S3.** Average (SD) correlation estimated between breeding values predicted for traditional (TL) and functional (FL) longevity using the complete data and all other scenarios, considering all ages (i.e., from 2 to 15 years) togeter and the design of 55,000 cows with uncensored records.

| **Longevity** | **^1^Scenario** | **Proportion of censored/penalized data** | | | |
| --- | --- | --- | --- | --- | --- |
| **indicator** |  | **20%** | **40%** | **60%** | **80%** |
| **TL** | **CEN** | 0.87 (0.01)a | 0.86 (0.01)a | 0.83 (0.01)b | 0.79 (0.02)c |
|  | **PENxm1** | 0.74 (0.03)d | 0.64 (0.06)e | 0.59 (0.05)f | 0.54 (0.07)g |
|  | **PENxm2** | 0.74 (0.03)d | 0.64 (0.05)e | 0.58 (0.07)f,g | 0.54 (0.09)g |
|  | **PENxm3** | 0.73 (0.04)d | 0.64 (0.05)e | 0.59 (0.06)f | 0.54 (0.08)g |
| **FL** | **CEN** | 0.89 (0.02)a,b | 0.86 (0.01)d | 0.84 (0.01)e | 0.82 (0.01)f |
|  | **PENxm1** | 0.90 (0.03)a | 0.89 (0.01)a,b | 0.88 (0.02)b,c | 0.87 (0.02)c,d |
|  | **PENxm2** | 0.88 (0.01)b,c | 0.88 (0.01)b,c | 0.87 (0.01)c,d | 0.86 (0.03)d |
|  | **PENxm3** | 0.89 (0.01)a,b | 0.87 (0.01)c,d | 0.86 (0.02)d | 0.87 (0.02)c,d |

**^1^**Scenarios included different proportions of censored data (CEN); penalized data assuming that all cows with censored records were culled one year after their last reported calving (PENxm1); penalizing data assuming that only cows with censored records older than nine years were culled one year after their last reported calving (PENxm2); and penalizing data assuming that cows with censored records older than nine years were culled one year after their last reported calving, while cows younger than nine years were culled two years after their last reported calving (PENxm3). Different letters, within each longevity indicator, show significant difference (p‐value <0.05) between values. Averages (and SD) shown here were obtained over the years and three replicates.
